# Editorial: Lacanian Psychoanalysis

**DOI:** 10.3389/fpsyg.2020.00192

**Published:** 2020-02-19

**Authors:** Gertrudis Van de Vijver, Jos de Kroon, Rémy Potier

**Affiliations:** ^1^Philosophy and Moral Science, Ghent University, Ghent, Belgium; ^2^Independent Researcher, Eindhoven, Netherlands; ^3^Paris Diderot University, Paris, France

**Keywords:** psychoanalysis, Lacan, Freud, drive, truth, speaking

The tricky thing about knowing is that one not just wishes to know, it is that one wishes to know, so to speak, for real. No kidding, the real-real, not the fake-real, that is what science, and yes, also psychoanalysis, are after. Certainly, the modern sciences were driven by the Greek ideal of being proven right in name of the truth—and not just, like the sophist, by the simple fact of wanting to be right. And the sciences of today are just on the same track, no matter how much relativity they are willing to take on board.

All this is a matter of speaking, of course, and psychoanalysis, and certainly Lacan's return to Freud, time and again confront us exactly with that: from the moment we speak, we, human subjects, are into the issue of truth, that is, into the issue of truth-seeking, inevitably and often quite desperately so. But then, psychoanalysis can reveal, perhaps more than any other discourse today, the decoys at play in this truth-seeking business, as well as the eagerness with which we are all inclined to forget about them, as if truth must be there, somewhere, somehow, even if only a little bit, just a little bit, …

In psychoanalysis, it is Lacan who most insistently stresses that the drive to know is what pulls us into subjectivity. And the fact that he is known to be most difficult of access, is perhaps related to that: the question for him is not to tell how things really are—how could he, after all?—it is to have us, all of us, one by one, circle around the structural impossibility that marks us as speaking beings, and to have us, all of us, one by one, address the question of what it means for us to have wanted and to continue to want to know. To attempt to overcome this structural impossibility is what makes us suffer, as it is what makes up our pleasure and enjoyment. In this structural impossibility lies our whole possibility.

This small introductory note is meant to invite the readers of this special issue on Lacanian Psychoanalysis to look upon the contributions from this methodological angle: the point is not to approach them against the background of a possible “real-real” truth, it is to consider them, each in their own way, clinically, theoretically, philosophically, as potential small eye openers…*Clinically*, for the occasions where the overall “wanting to cure”—a scandal, as Artonin Artaud reminds us—risks to overrule the very singular way speaking beings have of traveling in subjectivity, of inhabiting it by finding their very own and proper way of dying, as Freud so poignantly states in “Beyond the pleasure principle.” *Theoretically*, for the occasions where the sciences—in this regard it are most of the time the neurosciences—are invoked to bring on the “objective” authority, the “real” truth, and where not much room is left, except in the defensive mode, for a diverging voice, or a voice at all. *Philosophically*, for the occasions where the truth risks to pass only under the cover of an objectivity that speaks for itself, leading to an objectivism that can only side with a subjectivism that is as homeless and voiceless as it is itself.

This special issue contains 12 contributions.

Within the **“Hypothesis and Theory”** section, we have 5 contributions:

The contribution of Feyaerts and Vanheule, “The Logic of Appearance: Dennett, Phenomenology and Psychoanalysis,” deals with Daniel Dennett's take on the neurosciences, psychoanalysis, and contemporary phenomenology. It is the logic of appearance, stemming from Lacanian psychoanalysis, that serves here as a wager to confront and assess ideas about the first person perspective, consciousness, and issues of naturalization.

Potier and Putois start in “A Lacanian Approach to Medical Demand, With a Focus on Pediatric Genetics: A Plea for Subjectivization” from clinical experience in pediatric genetics. They take Lacan's remarks on the unconscious demand as a source of inspiration, and address the question in what sense medical demand, with healing and curing as its objects, can be viewed as a special instance of it—medical demand as an instance of transference, i.e., as an instance of seeking knowledge on the cause of one's desire. The notion of implicit demand is especially highlighted here, in line with the work of Raimbault, in turn inspired by Lacan, and this allows the authors to suggest that it might be interesting to supplement the medical perspective of curing and healing by what the “family myth” presents in relation to guilt and disease.

From a more historical angle, Razon et al. then give, in “The Lacanian Concept of Cut in Light of Lacan's Interactions with Maud Mannoni,” a detailed explanation on how Maud Mannoni's clinical experiences fed Lacan's theorizing of the concept of the cut and the symbolic operation through which the object *a* is produced.

In “The Mark, the Thing, and the Object: On what Commands Repetition in Freud and Lacan” Van de Vijver et al.'s “Beyond the pleasure principle,” the compulsion to repeat and Lacan's commentary on it. For Lacan the compulsion to repeat articulated in terms of jouissance, is a genuine break with the pleasure principle. Over and again Lacan stresses that repetition is the basis of subjectivity, not intentionality. Man is driven to repeat what was structurally missed and not be guided by what brings satisfaction to his needs.

Westphal and Lamote discuss in “The Clinic of Identifications in the Different Processes of Metamorphosis Into Woman” the function of identification of the unconscious subject in relation to the body, the body image, and the other. Clinical examples and examples out of the literature bring the authors to sustain that transsexualism, and metamorphosis into woman, can be considered as a way to problematize the relation to the body and to the other—a new opportunity and a new challenge in light of the changed legal context.

In the **“Conceptual Analysis”** section we have 4 contributions:

The relation between psychoanalysis and neuroscience is the subject of Aguiar's “The ‘Real Without Law’ in Psychoanalysis and Neurosciences.” Lacan's adage “The real of psychoanalysis is without law” apparently contrasts sharply with what the real is for science, i.e., according to Lacan, entirely governed by laws. Neither Lacan nor Freud defended an incompatibility between psychoanalysis and science, and the latest discoveries in neuroscience seem to bring them closer, in as far namely that contingency (“the real without law”) plays an ever more important role in the sciences.

Dimitriadis explores in “The Psychoanalytic Concept of Jouissance and the Kindling Hypothesis” the relation between Lacan's effort to articulate the concept of jouissance, current researches in the neurosciences, and clinical phenomena that witness to the fact that jouissance becomes “kindled,” i.e., escapes the control of the symbolic process. He suggests that the process of kindling can have a destructive effect, also in the brain, that he proposes to coin as a “psychosomatic disease of the brain.”

In “The Individual and the Collective: Sociological Influences on Lacan's Concept of the Relation Subject—Other,” Schrans gives a large exposition of the development of the family in Western and other societies elaborated by thinkers as Durkheim, Mauss, and Lévi-Strauss. It is shown how Lacan was influenced by these sociologists, how he changed his standpoint from a principle of an imaginary identification in the family to a concept in which the subject is structured in a symbolic system.

Vulnerability is the main concept under study in “Language and Vulnerability—A Lacanian Analysis of Respect” by Laufer and Santos. The article discusses the way in which Lacanian psychoanalysis allows to take into account the vulnerability of the subject, without reducing it thereby to a victim. Underlying here is the issue of the identity of the subject.

In the **“Perspective article”** section we have 2 contributions:

Lepoutre et al. give in “The Lacanian Concept of Paranoia: An Historical Perspective” an outline of Lacan's concept of paranoia that he considered as the “resistant nucleus” of psychosis in contrast with schizophrenia. Opposite to the neo-Kraepelinian approach in the DSM-enterprise where paranoia as a concept loses of its importance, Lacan puts it in the center of his theory on psychosis. The authors plea for a reinvestment of Lacan's concept of paranoia because of its subtleties and theoretical potentiality.

Ouvry informs us in “Lacan and Adolescence: The Contemporary Clinic of the ‘Sexual Non-rapport’ and Pornography” on the “Sexual Non-rapport” and pornography among adolescent boys. He explores two clinical phenomena and their risk of pathological fixation: pornography and conspiring thinking. Two aspects of the same principle are at stake: “seeing it” [ç*a voir*] and “knowledge” [*savoir*] applied to sexuality (pornography) and to truth (conspiracy theories). In both cases there is a denial of the recognition of the “other jouissance.”

In the “**Opinion Article**” section, we have 1 contribution:

De Battista deals in on “Lacanian Concept of Desire in Analytic Clinic of Psychosis” with Lacan's concept of desire in psychosis. She discusses the relative absence of references to the concept of psychotic desire in Lacan scholarship, and takes up the challenge of attempting to reconsider it in ways other than departing from the concept of the Name-of-the-Father.

## Author Contributions

GV is the main author of this editorial, followed by JK and RP.

### Conflict of Interest

The authors declare that the research was conducted in the absence of any commercial or financial relationships that could be construed as a potential conflict of interest.

